# Neuromodulation als vierte Säule in der Depressionsbehandlung: Ein Positionspapier des DZPG

**DOI:** 10.1007/s00115-026-01999-8

**Published:** 2026-08-05

**Authors:** Sarah Kayser, Beatrix Barth, Malek Bajbouj, Ann-Christine Ehlis, Martin F. Gerchen, Ahmed A. Karim, Joan Koti, Nils B. Kroemer, Fritz-Georg Lehnhardt, Thomas Nickl-Jockschat, Michael A. Nitsche, Marie-Luise Otte, Christian Plewnia, Alexander Sartorius, Stefan R. Schweinberger, Julia Siodmiak, Surjo R. Soekadar, Giovanni Vardiero, Ulrike Vogelmann, Tino Zaehle, Ulf Ziemann, David Zilles-Wegner, Alireza Gharabaghi, Andreas Fallgatter

**Affiliations:** 1 Klinik für Psychiatrie und Psychotherapie, Tübingen Center for Mental Health (TüCMH), Universitätsklinikum Tübingen, Calwerstraße 14, 72076 Tübingen, Deutschland; 2 Standort Tübingen, Deutsches Zentrum für Psychische Gesundheit (DZPG), Tübingen, Deutschland; 3 LEAD Graduate School & Research Network, Universität Tübingen, Tübingen, Deutschland; 4 Klinik und Hochschulambulanz für Psychiatrie, Charité Universitätsmedizin Berlin, Klinik für Psychiatrie und Psychotherapie Campus Benjamin Franklin, Berlin, Deutschland; 5 Standort Berlin-Potsdam, Deutsches Zentrum für Psychische Gesundheit (DZPG), Berlin, Potsdam, Deutschland; 6 Zentralinstitut für Psychische Gesundheit, Universität Heidelberg/Medizinische Fakultät Mannheim, Mannheim, Deutschland; 7 Standort Mannheim-Heidelberg-Ulm, Deutsches Zentrum für Psychische Gesundheit (DZPG), Mannheim, Heidelberg, Ulm, Deutschland; 8 Klinik für Psychiatrie und Psychotherapie, TUM Klinikum Rechts der Isar, Technische Universität München, München, Deutschland; 9 Standort München-Augsburg, Deutsches Zentrum für Psychische Gesundheit (DZPG), München, Augsburg, Deutschland; 10 Klinik für Psychiatrie, Universität Bonn, Bonn, Deutschland; 11 Klinik und Poliklinik für Psychiatrie und Psychotherapie, Klinikum der Universität zu Köln, Köln, Deutschland; 12 Klinik für Psychiatrie, Psychotherapie und Psychosomatik, Evangelisches Krankenhaus Bergisch Gladbach, Bergisch Gladbach, Deutschland; 13 Klinik und Poliklinik für Psychiatrie und Psychotherapie, Otto-von-Guericke-Universität Magdeburg, Magdeburg, Deutschland; 14 Department of Neuroscience and Pharmacology, Carver College of Medicine, University of Iowa, Iowa, IA USA; 15 Iowa Neuroscience Institute, University of Iowa, Iowa, IA USA; 16 Department of Psychiatry, Carver College of Medicine, University of Iowa, Iowa, IA USA; 17 Center for Behavioral Brain Sciences (CBBS), Magdeburg, Deutschland; 18 Standort Halle-Jena-Magdeburg, Deutsches Zentrum für Psychische Gesundheit (DZPG), Halle, Jena, Magdeburg, Deutschland; 19 Abteilung Psychologie und Neurowissenschaften, Leibniz-Institut für Arbeitsforschung, Dortmund, Deutschland; 20 Bielefeld University, University Hospital OWL, Evangelisches Klinikum Bethel, Universitätsklinik für Psychiatrie und Psychotherapie, Bielefeld, Deutschland; 21 Standort Bochum-Marburg, Zentrum für Psychische Gesundheit (DZPG), Bochum, Marburg, Deutschland; 22 Klinik für Allgemeine Psychiatrie, Department für Psychosoziale Medizin, Prävention und Familiengesundheit, Universität Heidelberg, Heidelberg, Deutschland; 23 Klinik für Psychiatrie und Psychotherapie, Zentralinstitut für Seelische Gesundheit, Mannheim, Deutschland; 24 Klinik für Psychiatrie und Psychotherapie, Campus Charité Mitte, Charité – Universitätsmedizin Berlin, Berlin, Deutschland; 25 Klinik für Neurologie, Otto-von-Guericke Universität Magdeburg, Magdeburg, Deutschland; 26 Center for Intervention and Research on adaptive and Maladaptive Brain Circuits Underlying Mental Health (CIRC), Jena-Magdeburg-Halle, Magdeburg, Deutschland; 27 Department of Neurology & Stroke, and Hertie Institute for Clinical Brain Research, Universität Tübingen, Tübingen, Deutschland; 28 Klinik für Psychiatrie und Psychotherapie, Universitätsmedizin Göttingen, Göttingen, Deutschland; 29 Institut für Neuromodulation und Neurotechnologie, Universitätsklinikum Tübingen, Tübingen, Deutschland; 30 Zentrum für Bionic Intelligence Tübingen Stuttgart (BITS), Tübingen, Deutschland

**Keywords:** Depressive Störung, Neuromodulation, Elektrokonvulsionstherapie, Transkranielle Magnetstimulation, Therapieresistente Depression, Depressive disorder, Neuromodulation, Electroconvulsive therapy, Transcranial magnetic stimulation, Treatment-resistant depression

## Abstract

**Hintergrund:**

Depressive Störungen gehen mit hoher Krankheitslast einher; ein relevanter Anteil der Patient:innen spricht trotz leitliniengerechter Pharmako- und Psychotherapie unzureichend an, insbesondere bei therapieresistenter Depression (TRD). Neuromodulation etabliert sich als vierte Säule der Depressionsbehandlung und umfasst Elektrokonvulsionstherapie (EKT), repetitive transkranielle Magnetstimulation (rTMS), Vagusnervstimulation (VNS), transkranielle elektrische Stimulation (tES), Magnetkonvulsionstherapie (MKT), tiefe Hirnstimulation (THS) und Neurofeedback (NF). Trotz teilweiser gut belegter Wirksamkeit sind diese Verfahren bislang nicht ausreichend in die Regelversorgung integriert. Ziel ist die Darstellung der Evidenzlage und zentraler Implementierungsbarrieren.

**Material und Methoden:**

Narrative Auswertung klinischer Studien, Metaanalysen und Versorgungsdaten zu Wirksamkeit, Sicherheit und kognitiver Verträglichkeit.

**Ergebnisse:**

Für EKT besteht die beste Evidenz hinsichtlich Akut- und Erhaltungstherapie schwerer depressiver Störungen; rTMS ist wirksam und kognitiv gut verträglich. VNS kann eine additive Langzeitoption bei TRD darstellen. Für tES werden kleine, heterogene Effekte berichtet. MKT und THS bleiben experimentell; für NF fehlen konsistente Wirksamkeitsnachweise. Zentrale Barrieren sind teilweise heterogene Protokolle, unzureichende Finanzierungsstrukturen, insbesondere im ambulanten Bereich, und fehlende Qualifikationsstandards.

**Schlussfolgerungen:**

Neuromodulation sollte systematisch in gestufte Versorgungsmodelle integriert werden. Standardisierte Protokolle und Registerforschung sind erforderlich, um eine strukturierte und nachhaltige Implementierung neuromodulatorischer Verfahren in die klinische Versorgung zu ermöglichen.

## Abstract (en)

**Background:**

Depressive disorders are associated with a high burden of disease; a substantial proportion of patients show insufficient response despite guideline-based pharmacotherapy and psychotherapy, particularly in treatment-resistant depression (TRD). Neuromodulation has become established as a fourth pillar in the treatment of depression and includes electroconvulsive therapy (ECT), repetitive transcranial magnetic stimulation (rTMS), vagus nerve stimulation (VNS), transcranial electrical stimulation (tES), magnetic seizure therapy (MST), deep brain stimulation (DBS) and neurofeedback (NF). Despite partly well-established efficacy, these interventions remain insufficiently integrated into routine clinical care. The aim of this paper is to summarize the current evidence and to analyze key barriers to implementation.

**Material and methods:**

Narrative review of clinical studies, meta-analyses, and care data on efficacy, safety and cognitive tolerability.

**Results:**

The strongest evidence for both acute and maintenance treatment of severe depressive disorders is for ECT; rTMS is effective and cognitively well tolerated and VNS can represent an additive long-term option in TRD. For tES, small and heterogeneous effects have been reported, MST and DBS remain experimental and for NF consistent evidence of efficacy is lacking. Key barriers include partly heterogeneous protocols, insufficient funding structures, particularly in the outpatient setting and the absence of standardized qualification requirements.

**Conclusion:**

Neuromodulation should be systematically integrated into staged-care models. Standardized protocols and registry-based research are needed to enable the structured and sustainable implementation of neuromodulatory interventions in clinical care.

Neuromodulation gewinnt als vierte Säule der Depressionsbehandlung neben Pharmako‑, Psycho- und Sozialpsychiatrie zunehmend an Bedeutung, insbesondere bei therapieresistenter Depression. Das Positionspapier ordnet etablierte und experimentelle Verfahren anhand der aktuellen Evidenz zu Wirksamkeit, Sicherheit und kognitiver Verträglichkeit ein und diskutiert ihren Stellenwert in gestuften Behandlungsmodellen. Darüber hinaus werden zentrale Implementierungsbarrieren adressiert und Empfehlungen zu Standardisierung, Qualifikation, Finanzierung und Versorgungsintegration formuliert.

Depressive Störungen stellen trotz evidenzbasierter Pharmako- und Psychotherapie weiterhin eine erhebliche klinische und versorgungspolitische Herausforderung dar. Neuromodulatorische Verfahren gewinnen insbesondere bei TRD zunehmend an Bedeutung, werden jedoch selten eingesetzt und unterschiedlich implementiert. Vor diesem Hintergrund besteht Bedarf an einer strukturierten Einordnung ihres klinischen Stellenwerts sowie der damit verbundenen Anforderungen an Qualitätssicherung, Qualifikation und Versorgungsstrukturen.

## Einleitung

### Krankheitslast und therapeutische Limitationen depressiver Störungen

Depressive Störungen verursachen eine hohe Krankheitslast, gehen mit Chronifizierung und funktionellen Einschränkungen einher [16]. Trotz evidenzbasierter Pharmako- und Psychotherapie bleibt ein relevanter Anteil der Betroffenen unzureichend behandelt, insbesondere bei TRD [13]. Dies unterstreicht den Bedarf an ergänzenden Behandlungsoptionen.

### Neuromodulation: Verfahren, Versorgungsstatus und Barrieren

Neuromodulation etabliert sich als vierte Säule der Depressionsbehandlung und umfasst EKT, rTMS, VNS sowie experimentelle Verfahren wie tES, MKT, THS und NF.

EKT und rTMS sind in Deutschland Bestandteil der klinischen Versorgung; die EKT ist seit Jahrzehnten Teil der Regelversorgung [1]. Für rTMS und implantierbare VNS bestehen CE-zertifizierte Medizinprodukte. tES werden weiterhin evaluiert, während MKT, THS und NF überwiegend im spezialisierten Kontext eingesetzt werden.

Trotz hoher Evidenz sind etablierte neuromodulatorische Verfahren strukturell nicht bedarfsgerecht implementiert. Hauptbarrieren betreffen Zugang, Vergütung, Standardisierung und Qualifikation.

### Zielsetzung des Positionspapiers

Dieses Positionspapier fasst die Evidenz zu Wirksamkeit, Sicherheit und kognitiver Verträglichkeit neuromodulatorischer Verfahren in der Akut‑, Erhaltungs- und Rückfallbehandlung zusammen, mit besonderem Fokus auf therapieresistente Depression.

Es ordnet etablierte (EKT, rTMS, VNS) und experimentelle Verfahren (tES, NF, MKT, THS) nach Evidenz und klinischer Etabliertheit ein und leitet aus bestehenden Barrieren in Regulierung, Finanzierung, Standardisierung und Qualifikation konkrete Handlungsempfehlungen ab.

Ziel ist die strukturelle Integration neuromodulatorischer Verfahren in evidenzbasierte Versorgungsmodelle sowie die Förderung ihrer qualitätsgesicherten Implementierung in spezialisierten Netzwerken.

## Elektrokonvulsionstherapie (EKT)

Die EKT gilt als wirksamstes und zugleich sicheres Neurostimulationsverfahren bei TRD. Unter Kurznarkose wird durch kurze Stromimpulse ein kontrollierter epileptischer Anfall induziert. Für wahnhafte Depression und TRD wird sie in der S3-Leitlinie Unipolare Depression sowie in der DGPPN-Stellungnahme 2022 mit höchstem Empfehlungs- und Evidenzgrad ausgewiesen. Auch in der S3-Leitlinie Bipolare Störungen erhält sie einen hohen Empfehlungsgrad. Die Indikationsstärke nimmt mit Schweregrad und Lebensalter zu. Ihre ausgeprägte Akutwirksamkeit, gute Verträglichkeit sowie der belegte Nutzen hinsichtlich Mortalitäts- und Suizidreduktion sind konsistent dokumentiert [5]. Nach der Indexserie wird bei schweren rezidivierenden oder psychotischen Verläufen eine Erhaltungs-EKT empfohlen (typischerweise für 6–24 Monate), insbesondere auf Wunsch des Patienten oder falls ein medikamentös/psychotherapeutischer Rückfallschutz nicht wirksam oder nicht verträglich ist.

Die EKT weist ein hohes Sicherheitsprofil auf. Häufigste Nebenwirkungen sind vorübergehende kognitive Einschränkungen, meist reversibel innerhalb von 2 bis 3 Wochen [15]. Ihr Ausmaß hängt von patientenbezogenen Faktoren (Alter, Residualsymptomatik, Komedikation) sowie vom Stimulations- und Anästhesieprotokoll ab.

Die Vergütung erfolgt überwiegend stationär oder teilstationär über PEPP-Zusatzentgelte (ZP73.01/73.02). Als Entgelt für die EKT stellt der Einheitliche Bewertungsmaßstab (EBM) die Gebührenordnungsposition (GOP) 21330 „Konvulsionsbehandlung“ als „Analogziffer“ zur Verfügung, was mit derzeit 94 Punkten oder 11,98 € aber eine echte Barriere für eine ambulante Implementierung darstellt. Standardisierte Verfahrensanweisungen ermöglichen eine qualitätsgesicherte Anwendung [14]. Routinedaten zeigen eine steigende, jedoch weiterhin niedrige Nutzung in Deutschland (0,69/10.000 Einwohner/Jahr); nur etwa die Hälfte der psychiatrischen Kliniken bietet EKT an. Neben strukturellen und abrechnungsbezogenen Hürden bestehen organisatorische und teils kulturelle Barrieren. Seit 2020 ist die EKT Bestandteil der Facharztweiterbildung, was langfristig zu einer Verbesserung der Versorgung beitragen dürfte.

## Transkranielle Magnetstimulation (TMS)

Die rTMS ist ein etabliertes, evidenzbasiertes Verfahren zur Behandlung der TRD. Ihre Wirksamkeit ist durch zahlreiche randomisiert-kontrollierte Studien und Metaanalysen belegt und zeigt eine signifikante Überlegenheit gegenüber Scheinstimulation, mit Responseraten von 30–50 % und Remissionsraten von 20–45 % [3]. Die moderaten Effektstärken (Cohen’s d ≈ 0,4–0,6) entsprechen pharmakologischen Zweitlinienstrategien bei günstigerem Nebenwirkungsprofil.

Am besten untersucht ist die hochfrequente (≥ 10 Hz) Stimulation des linken dorsolateralen präfrontalen Kortex (dlPFC), die über die Modulation frontolimbischer Netzwerke wirkt [9]. Die intermittierende Theta-Burst-Stimulation (iTBS) erreicht bei deutlich verkürzter Protokolldauer vergleichbare Response- und Remissionsraten, was zur FDA-Zulassung beider Verfahren führte.

Leitlinien bewerten die hochfrequente linkspräfrontale rTMS mit höchstem Evidenzgrad (Level I; Empfehlungsgrad A) als Zweitlinien- oder Add-on-Therapie. Das Nebenwirkungsprofil ist insgesamt günstig: häufig mild-transiente Kopfschmerzen oder lokale Beschwerden; das Anfallsrisiko liegt bei sachgerechter Anwendung unter 0,1 %. Kognitive Beeinträchtigungen treten nicht auf. Vielmehr zeigen Studien bei kognitiv beeinträchtigten Patient:innen unter hochfrequenter dlPFC-Stimulation eine Verbesserung der globalen Kognition [9]. rTMS ist heute eine wichtige Komponente im multimodalen Behandlungspfad der Depression.

## Vagusnervstimulation (VNS)

Die implantierbare VNS ist ein operatives Verfahren zur Behandlung der TRD. Über einen subkutanen Stimulator wird der linke Nervus vagus intermittierend stimuliert und limbisch-kortikale Netzwerke moduliert [11].

Randomisierte Studien mit kurzer Nachbeobachtungsdauer zeigen teils keine signifikanten Unterschiede, während Langzeitstudien anhaltende Effekte berichten. Mehrjährige Kohorten weisen Responseraten von 30–50 % und Remissionsraten von 20–40 % aus, assoziiert mit reduzierten Hospitalisierungen und Suizidraten [12]. Das Sicherheitsprofil ist günstig; häufige Nebenwirkungen wie Heiserkeit oder Husten sind meist mild und dosisabhängig. Die Gesamtevidenz wird aufgrund begrenzter RCT-Daten als moderat bewertet, weshalb eine Leitlinienempfehlung bislang fehlt.

Die transkutane VNS (tVNS) zeigt in kleinen Studien moderate Effekte, gilt jedoch weiterhin als experimentell [17].

## Transkranielle elektrische Stimulation (tES)

Die tES umfasst Gleichstrom- (tDCS) und Wechselstromverfahren (tACS) zur Modulation kortikaler Erregbarkeit. Bei Depression wird überwiegend anodale tDCS über dem dlPFC eingesetzt.

Metaanalysen berichten kleine, heterogene Effekte mit Responseraten von etwa 20–30 % [2]. Die Wirksamkeit scheint interindividuell variabel und abhängig von anatomischen sowie physiologischen Faktoren. tES ist gut verträglich, Nebenwirkungen sind meist mild und transient.

Trotz CE-Zweckbestimmung ist das Verfahren derzeit nicht als Standardtherapie etabliert. Regulatorische Einstufung (MDR Klasse III), fehlende Erstattungsstrukturen und heterogene Protokolle limitieren die Implementierung.

## Magnetkonvulsionstherapie (MKT)

Die MKT ist ein konvulsives Verfahren, bei dem unter Narkose mittels Magnetstimulation ein therapeutischer, epileptischer Anfall ausgelöst wird. Eine Metaanalyse berichtet eine gute Akutwirksamkeit bei günstigerem kognitivem Nebenwirkungsprofil im Vergleich zur EKT [10]. Randomisierte Nichtunterlegenheitsstudien fehlen bislang; die Evidenz zur Erhaltungsbehandlung ist begrenzt [4]. Die MKT wird überwiegend im Studienkontext angewandt.

Perspektivisch könnte sie eine Alternative für Patient:innen mit TRD darstellen, insbesondere bei erhöhter kognitiver Vulnerabilität, vorausgesetzt standardisierte Protokolle und multizentrische Studien.

## Tiefe Hirnstimulation (THS)

Die THS ist ein invasives Verfahren zur Behandlung schwerer TRD, bei dem subkortikale Zielregionen kontinuierlich stimuliert werden (u. a. SCC, VC/VS, NAcc, slMFB). Langzeitstudien berichten Ansprechraten von 40–60 % und Remissionsraten um 25 % [8]. Randomisierte Studien zeigen jedoch keine konsistente Überlegenheit gegenüber Scheinstimulation [6]. Bildgebungsdaten und Traktografieanalysen legen eine netzwerkbasierte Zieldefinition nahe; adaptive Stimulationsansätze werden untersucht. Das Sicherheitsprofil ist insgesamt günstig, schwerwiegende Komplikationen sind selten und kognitive Funktionen bleiben erhalten.

Aufgrund begrenzter RCT-Evidenz erfolgt der Einsatz derzeit überwiegend im Studienkontext.

## Neurofeedback (NF)

Das NF ermöglicht die Selbstregulation neuronaler Aktivität mittels EEG- oder fMRT-basierter Echtzeitrückmeldung. Externe Stimulation erfolgt nicht.

Studien berichten symptomatische Verbesserungen bei Depressionen, die Evidenz ist jedoch heterogen und häufig auf kleine Stichproben begrenzt [7, 18]. Langzeiteffekte sind unzureichend untersucht. NF weist ein günstiges Sicherheitsprofil auf, gilt jedoch mangels standardisierter Protokolle und robuster RCT-Daten weiterhin als experimentell.

## Empfehlungen

### Stellenwert der Neuromodulation im Behandlungspfad

Neuromodulatorische Verfahren sollen als Bestandteil gestufter Behandlungsmodelle bei Depression berücksichtigt werden, insbesondere bei Nichtansprechen auf leitliniengerechte Therapie und bei TRD. Die Auswahl sollte sich an Evidenz, Schweregrad, kognitiver Vulnerabilität, Komorbiditäten, Patient:innenpräferenz und Versorgungsrealität orientieren.

Die EKT stellt bei TRD den Goldstandard mit höchster Akutwirksamkeit dar und sollte frühzeitig erwogen werden. rTMS ist als evidenzbasierte, gut verträgliche Zweitlinien- bzw. Add-on-Therapie empfohlen. Die implantierbare VNS kann bei chronischer oder rezidivierender TRD als additive Langzeitoption erwogen werden.

### Umgang mit experimentellen Verfahren

Für tES, MKT, THS und NF besteht derzeit keine ausreichende Evidenz für eine breite Regelversorgung. Ihr Einsatz sollte primär im Studienkontext erfolgen. Erforderlich sind größere RCTs, standardisierte Protokolle und Daten zur Erhaltungstherapie.

### Qualität und Implementierung

Voraussetzung für eine qualitätsgesicherte Anwendung sind standardisierte Prozesse, definierte Qualifikationsanforderungen und strukturierte Aufklärung. Eine nachhaltige Implementierung erfordert angemessene Erstattungsmodelle, vernetzte Versorgungsstrukturen sowie den Ausbau spezialisierter Zentren und Fortbildungsangebote.

## Perspektiven

Weiterer Forschungsbedarf besteht insbesondere für multizentrische Register und randomisierte Studien zu Langzeiteffekten, Kosteneffektivität und Prädiktoren des Therapieansprechens. Zentrale Entwicklungen betreffen personalisierte Stimulationsansätze (z. B. konnektivitätsbasierte Ziele, neurophysiologische Marker, adaptive Protokolle) sowie belastbare Daten zur Erhaltungsbehandlung und Rückfallprophylaxe.

Verlaufsinstrumente, telemedizinische Nachsorge und KI-gestützte Analysen bieten zusätzliche Möglichkeiten zur Individualisierung.

Strukturell wird die Zukunft der Neuromodulation von einer engen Vernetzung von Forschung, Qualifikation und Versorgung abhängen, etwa durch spezialisierte Zentren und Register, um Evidenzgenerierung und klinische Implementierung nachhaltig zu verzahnen.

## Fazit für die Praxis

- Elektrokonvulsionstherapie (EKT) und repetitive transkranielle Magnetstimulation (rTMS) sind evidenzbasierte, klinisch etablierte Verfahren.
- Die Vagusnervstimulation (VNS) stellt eine Option für ausgewählte Patient:innen mit chronischer oder rezidivierender therapieresistenter Depression (TRD) dar.
- Für transkranielle elektrische Stimulation (tES), Magnetkonvulsionstherapie (MKT), tiefe Hirnstimulation (THS) und Neurofeedback (NF) ist die Evidenz derzeit begrenzt; ihr Einsatz sollte überwiegend im Studien- oder spezialisierten Kontext erfolgen.
- Die nachhaltige Implementierung neuromodulatorischer Verfahren erfordert Qualitätsstandards, strukturierte Weiterbildung, tragfähige Finanzierung und eine enge Verzahnung von Forschung und klinischer Versorgung.

## Data Availability

Alle dieser Arbeit zugrunde liegenden Daten sind in diesem Artikel enthalten.
